# E-cigarettes for Smoking Cessation: The Courage to Embrace the Science Around Nicotine Addiction

**DOI:** 10.1016/j.jacadv.2025.102106

**Published:** 2025-08-22

**Authors:** Reto Auer, Kali Tal, Stéphanie Baggio

**Affiliations:** a Substance Use Unit, Institute of Primary Health Care (BIHAM), University of Bern, Bern, Switzerland; b Centre for Primary Care and Public Health (Unisanté), Lausanne, Switzerland; c Laboratory of Population Health (#PopHealthLab), University of Fribourg, Fribourg, Switzerland

**Keywords:** e-cigarette, harm reduction, moral dilemma, nicotine, smoking, tobacco

**Figure:**
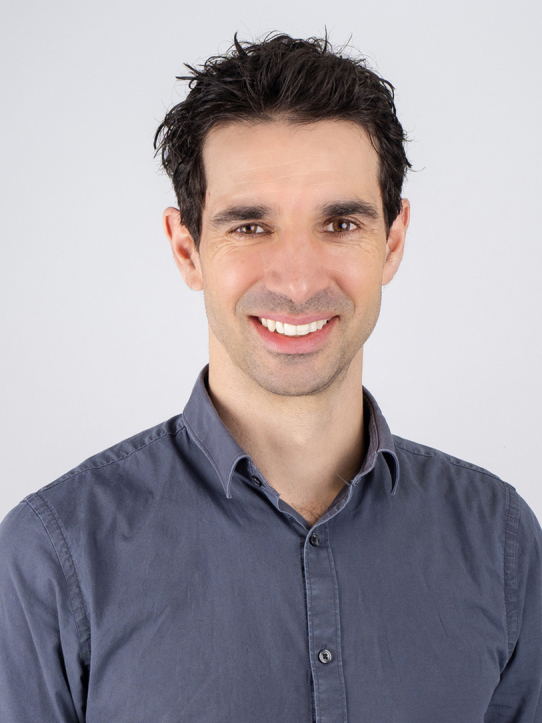

E-cigarettes—battery-powered devices that vaporize nicotine-containing liquid—quickly deliver nicotine to the brain through lung inhalation, giving users a “nicotine flash” that nicotine replacement therapy (NRT) patches or gum cannot match.^1^^,^^2^ A range of laboratory and clinical toxicologic analyses confirm that e-cigarette users are exposed to fewer and less inhaled toxins than tobacco smokers, which should reduce the well-documented harms associated with tobacco smoking, albeit with concerns related to health effects of heated chemical aerosols.^3^, ^4^, ^5^ But the very attributes that make e-cigarettes attractive for tobacco abstinence may encourage prolonged nicotine use and lower the barriers for adolescents and nicotine-naïve people who wish to experiment. We believe moral arguments on nicotine use explain why the authors of clinical guidelines have been reluctant to recommend e-cigarettes for tobacco smoking abstinence despite mounting high-quality evidence that e-cigarettes are an effective and safe option for short-term use as a smoking cessation aide.^1^ We encourage guideline developers to openly address moral dilemmas on nicotine use when incorporating the clear and convincing evidence provided by randomized controlled trials (RCTs) like that of Fillion et al, reported in this month’s JACC-Advances.^6^

## Results of the E3 trial at 12-month follow-up

E3 (Evaluating the Efficacy of e-Cigarette Use for Smoking Cessation) was a well-designed, rigorous RCT conducted across 17 centers in Canada. E3 compared the following three interventions: nicotine e-cigarettes (15 mg/mL) plus individual counseling; non-nicotine e-cigarettes (0 mg/mL) plus individual counseling; and individual counseling alone. The study included patients typically seen in clinics, who had failed to quit with other methods despite acquiring health conditions associated with tobacco smoking. The investigators took the pragmatic approach of focusing on tobacco smoking abstinence, whether or not e-liquids contained nicotine. Participants in the active groups were provided free e-cigarettes, which they could consume ad libitum; they were not instructed to gradually taper off e-cigarettes.

Results from E3’s 3-month follow-up (published in 2020) revealed the nicotine-containing e-cigarette group had higher tobacco cigarette abstinence rates than the control group.^7^ After 3 months, participants were told to return the study products. Those who wished to continue using e-cigarettes had to buy their own devices and e-liquids. The current report revealed outcomes up to 12-months. At 12-months, self-reported abstinence from cigarette smoking in the week before the outcome was assessed (validated by exhaled carbon monoxide) was still more than twice as high in the nicotine-containing e-cigarette group than in the control group. Non-nicotine e-cigarettes were similarly efficacious. Adverse events were generally mild and comparable between groups. The few serious adverse events (SAEs) were rigorously tracked, and SAEs were independently adjudicated by an independent, blinded Endpoints Evaluation Committee and supervised by an independent Data Safety and Monitoring Board.

## Contribution to the evidence base

Fillion et al should be commended for their high-quality methods, conduct, and interpretation of E3 trial results. They tested the efficacy and safety of e-cigarettes with methodology and rigor usually reserved for pharmaceutical products and met the reporting standards for RCT that test smoking cessation products.

E3 trial results at 3 months are included in the comprehensive Cochrane living systematic review on e-cigarettes for smoking cessation.^1^ The Cochrane review’s conclusions are clear: E-cigarettes enable higher smoking cessation rates than NRT. The results of the main comparison in the trial by Fillion et al, between nicotine e-cigarettes and usual care, aligned with Cochrane’s, where weighted risk ratio from pooling results of 5,024 participants across 9 studies on continuous tobacco smoking abstinence was 1.88 (95% CI: 1.56-2.25).

Pooled results from Cochrane (1,613 participants across 7 studies) suggest that people quitting tobacco might benefit from nicotine e-cigarettes compared to non-nicotine e-cigarettes (weighted risk ratio: 1.46 (95% CI: 1.09-1.96), whereas the E3 trial found tobacco cigarette cessation rates were similar in the nicotine and non-nicotine e-cigarettes groups.^1^ However, in the E3 trial, participants from all groups consumed nicotine in other forms and few used non-nicotine e-cigarettes after the 3-months follow-up. Also, by the 12-month visit, members of the non-nicotine group were more likely to use nicotine-containing e-cigarettes after 3 months than members of the control group. Although non-nicotine e-cigarettes may replace the haptic experience of tobacco cigarettes and be sufficient for some, pooled results from Cochrane suggest that nicotine e-cigarettes further increase tobacco smoking abstinence rates.^1^

Finally, Cochrane reports of an absence of evidence risk of SAE increases with e-cigarettes for smoking cessation; integrating E3’s results at 52 weeks should tighten the CIs on these estimates.^1^

## Challenges in implementing E3 trial results in clinical guidelines and clinical care

Although health care professionals are usually quick to prescribe and recommend new medications when well-designed RCTs reveal new medications to be twice as effective, they have not rushed to make the case for e-cigarettes. Despite the clarity of E3 trial results and their alignment with the Cochrane systematic review (27,235 participants across 88 studies; 47 studies reported the results of RCT like E3), few clinical guidelines describe e-cigarettes as an effective and safe method of smoking cessation. Instead, public health authorities and medical associations around the world take an active stance against e-cigarettes for smoking cessation.^8^ There is now enough data from RCT to show that nicotine-containing e-cigarettes are an effective and safer alternative to tobacco cigarettes at 12-months, so there must be another reason for hesitation. Beyond long-term safety concerns or challenging regulatory oversight of e-cigarettes in a rapidly evolving product landscape,^9^ we argue that hesitation is caused by moral dilemmas about nicotine use.

First, many clinicians, policymakers, and members of the public are reluctant to accept that tobacco smokers will continue using nicotine in some form, whether or not they abstain from tobacco cigarettes and advocate long-term nicotine abstinence as the only valid and safe goal. A focus on nicotine abstinence implies we tell patients who have so far failed to quit with NRT or other smoking cessation drugs to keep on trying.

Others accept that nicotine abstinence might be an unrealistic goal for many smokers, especially in the short term and propose an empathic and pragmatic harm reduction approach that focuses on tobacco smoking abstinence, with nicotine abstinence as a secondary, optional goal. Although some wait for long-term safety data before recommending e-cigarettes, others consider the extant data from the laboratory, 12-months follow-up data from RCT, and further observational data suffice.

We believe guideline developers could apply principles of shared decision-making to resolve this moral debate. Recommendations for treatment options could integrate patients’ preferences and values toward nicotine abstinence. Treatment algorithms could recommend clinicians ask their patients if they want to abstain from tobacco, and if they want to abstain from nicotine. If a tobacco smoker wants to abstain from tobacco but does not feel ready to abstain from nicotine, guidelines may recommend e-cigarettes as a treatment option. But if patients want to abstain from both tobacco and nicotine, guidelines could favor varenicline or cytisine.

Second, when highlighting the relative safety of e-cigarettes compared to ongoing tobacco smoking, scientists writing articles and clinicians counseling patients should acknowledge the risk that some adolescents and nonsmokers will mistake this harm reduction approach aimed at current tobacco smokers and use it to rationalize experimentation. This risk is increased when e-cigarette manufacturers push “safety” when they aggressively advertise e-cigarettes to young consumers. Unrestricted advertising and easy access increase the risk young experimenters will develop addiction to nicotine and be exposed to toxins released by e-cigarettes. Guidelines developers can address this moral dilemma with a two-pronged approach. Guidelines could draw on the extant literature from RCTs and include e-cigarettes as a valid smoking cessation aid for some smokers, while highlighting the need to inform adolescent nonsmokers that e-cigarettes may harm their health. Guideline authors should also advocate for regulating advertising and sales to youth.^8^

Patients interested in learning about evidence-based treatment options for smoking cessation must be informed of the potential benefits of e-cigarettes and the relative short-term safety of e-cigarettes when compared to tobacco cigarette smoking. They should also be informed of the likelihood of ongoing nicotine use and residual exposure to toxins through e-cigarettes use. To meet our ethical obligations to patients, we must neither dismiss evidence of the benefits of e-cigarettes, nor deny e-cigarettes to patients who are likely to benefit from them. But we must also be honest about the rate of ongoing nicotine use among smokers who quit with e-cigarettes and potential uptake of e-cigarettes by adolescents and nonsmokers.

We advocate for taking the hard road and facing these moral dilemmas. Based on data provided by Fillion et al and other researchers, guideline writers and clinicians should acknowledge the potential benefits of e-cigarettes for smoking cessation, and advocate against their uptake in populations likely to suffer more harms than benefits.

## Funding support and author disclosures

Dr Auer has received funding to test the efficacy, safety, and toxicology of e-cigarettes by 10.13039/501100001711Swiss National Science Foundation (grant number IICT_33IC30_173552 and 33IC30_213614), Swiss Tobacco Prevention Fund (grant number 19.017477), 10.13039/501100013362Swiss cancer research (grant number KFS4744-02-2019), and LungeZürich. All other authors have reported that they have no relationships relevant to the contents of this paper to disclose.
